# Proximal Femoral Replacement for Infected Non-union of the Proximal Femur With Adherent Femoral Artery: Surgical Challenges and Techniques to Minimize Vascular Risk

**DOI:** 10.7759/cureus.107733

**Published:** 2026-04-26

**Authors:** Prithviraj Deshmukh, Hisham Turjman, Kuntal Patel, Deepak Herlekar

**Affiliations:** 1 Trauma and Orthopaedics, University Hospitals of Morecambe Bay, Royal Lancaster Infirmary, Lancaster, GBR

**Keywords:** constrained liner, hip reconstruction, infected non-union, megaprosthesis, profunda femoris artery, proximal femoral replacement, proximal femur, vascular injury

## Abstract

Chronic infection and non-union of the proximal femur pose significant reconstruction challenges, where repeated surgical interventions can lead to bone loss and vascular adherence. The attachment of the profunda femoral artery (PFA) adds a substantial risk of intra-operative bleeding. We report a case of a 72-year-old female patient with ongoing pain and severe functional limitation after failed fixation complicated by chronic infection and subsequent excision arthroplasty. She underwent a proximal femur replacement (PFR) using a DePuy Synthes modular system with a constrained acetabular liner. Before the operation, infection control was achieved, and vascular mapping was done via CT angiogram, which suggested tethering of the PFA to the medial femoral cortex. Sharp dissection, vascular isolation with loops, and Doppler monitoring were all part of the intra-operative strategy used. This case report demonstrates that PFR can be an effective limb salvage option in the context of chronic infection and non-union of a proximal femoral fracture, and how operational success depends on careful pre-operative planning and precise surgical execution.

## Introduction

Infected non-unions present a difficult obstacle towards successful bone healing and require control of the infection [1]. Non-unions have a significant incidence of 1.9-10% among all fractures and are a result of the interplay between modifiable (obesity, smoking, malnutrition, etc.) and non-modifiable risk factors (open fractures, fractures with bone oss, etc.) [2,3]. These cases are characterized not only by loss of bone stock and prior implant failure, but also by significant soft tissue fibrosis and anatomical distortion from infection and repeated interventions [2]. Compromised blood supply contributes to non-union and promotes pathogen growth by their use of healthy bone for nutrition [2]. This pathophysiology is directly integrated in the mechanism of our report’s complication and brings to light the under-reported concern of adherence of major vascular structures to the surgical field (particularly the profunda femoral artery (PFA) in proximal femoral surgeries), which greatly increases the risk of intraoperative vascular injury.

Available data on the treatment of proximal femoral non-unions is limited, given the rarity of this complication [3]. Our report presents treatment modalities used, informative data on the complications, and the surgical challenges involved in such complex procedures that contribute to infected malunion.

## Case presentation

**Figure 1:**
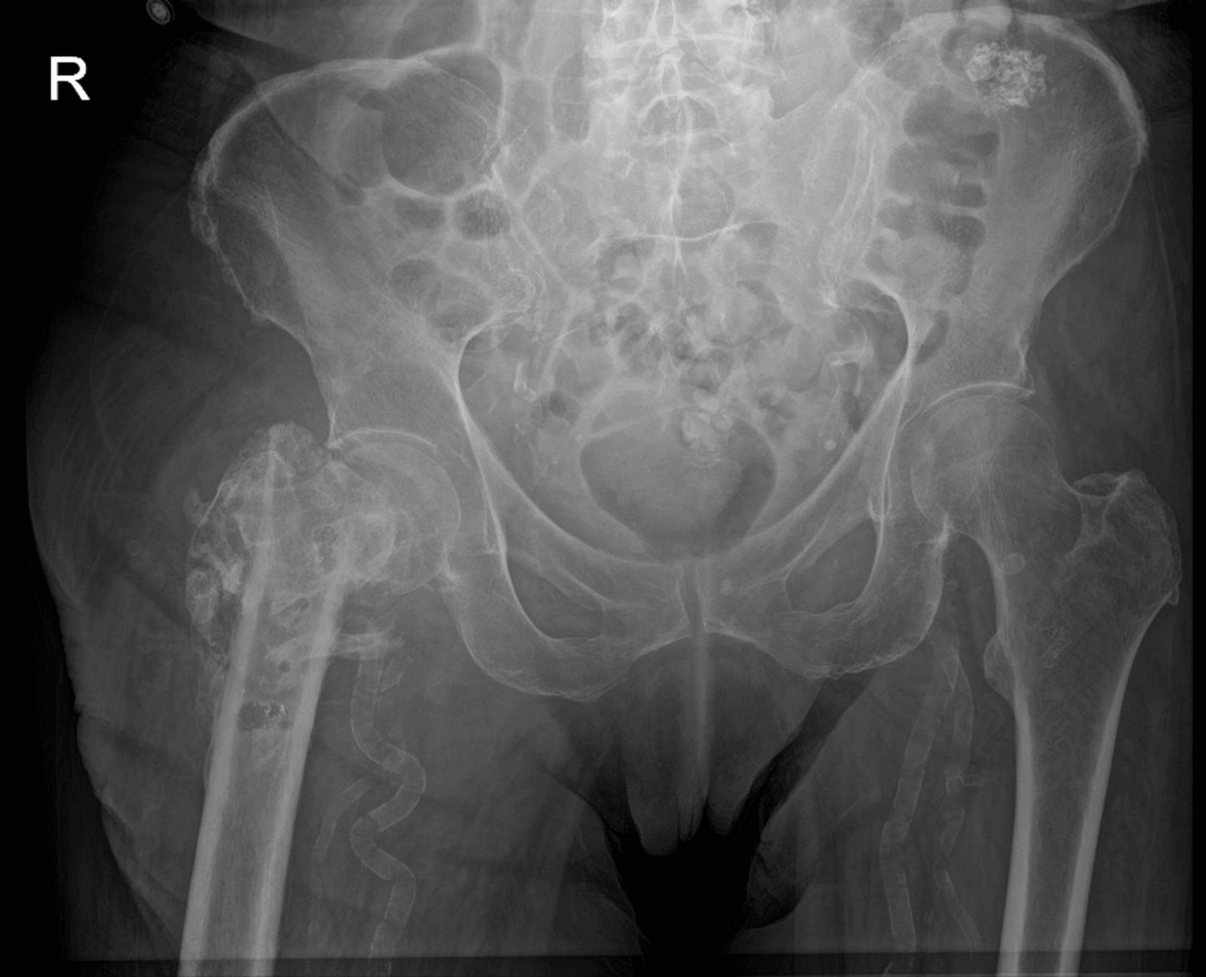
Pre-operative anteroposterior pelvis X-ray demonstrating right proximal femoral remodelling deformity An anteroposterior pelvic radiograph taken four months prior to surgery showing marked remodelling deformity of the right proximal femur with superior displacement of the distal fragment, consistent with chronic post-infective and post-fracture changes. No acute fracture or dislocation is evident. Mild degenerative changes are present within both hip and sacroiliac joints, with vascular calcification visible. The left hip joint remains preserved. These findings, in conjunction with persistent pain and mechanical instability, led to the decision to proceed with right proximal femoral replacement.

**Figure 2:**
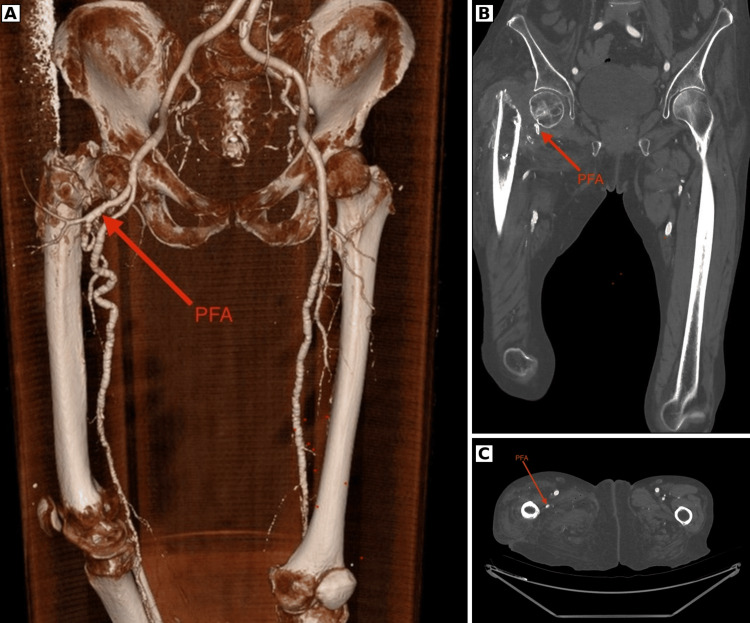
Pre-operative CT angiography demonstrating close relation of the profunda femoral artery to the proximal femur Pre-operative CT angiographic imaging demonstrating the vascular anatomy on the affected side. (A) 3D reconstruction showing the profunda femoral artery in close relation to the proximal femur. (B) Coronal image demonstrating the vessel adjacent to the medial proximal femur. (C) Axial image showing the vessel in close proximity to the proximal femur, supporting pre-operative planning. PFA, profunda femoral artery.

A 72-year-old female sustained a four-part intertrochanteric fracture of the right femur in October 2022 following a mechanical fall, initially treated with dynamic hip screw (DHS) fixation. Early postoperative wound discharge due to *Serratia marcescens* was managed with surgical washouts and antibiotics. The fracture failed to unite, complicated by recurrent infection with *Finegoldia magna*, necessitating implant removal in March 2024. Due to persistent non-union and infection (Figure 1), a Girdlestone excision arthroplasty was performed, significantly compromising her functional capacity. Following clinical, laboratory, and joint aspiration confirmation of infection quiescence by April 2025, CT angiography revealed adherence of the superficial and profunda femoral arteries to the medial femoral cortex (Figure 2). She was subsequently planned for proximal femoral replacement.

**Figure 3:**
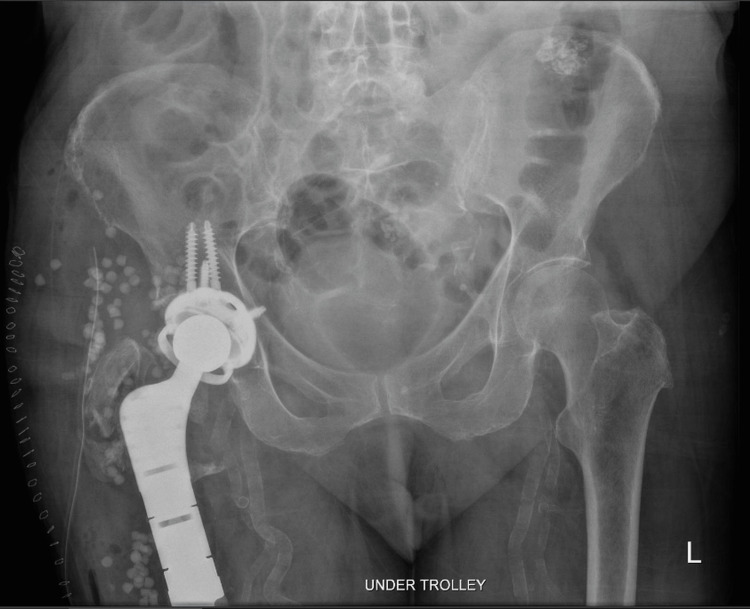
Immediate post-operative anteroposterior pelvis radiograph following right-sided cemented DePuy Synthes modular proximal femoral replacement An anteroposterior pelvis radiograph taken on the same day of surgery demonstrating a right-sided cemented DePuy Synthes modular proximal femoral replacement with an uncemented DePuy Synthes Pinnacle acetabular cup and constrained liner. The femoral stem is well-seated within the cement mantle, and multiple screws secure the acetabular reconstruction cage. The greater trochanter was preserved and reattached through the prosthesis to restore abductor integrity. Prophylactic cerclage wiring was applied around the bone-implant interface for additional construct stability. Antibiotic-loaded STIMULAN beads are visible around the implant site. The right hip joint remains native and intact.

**Figure 4:**
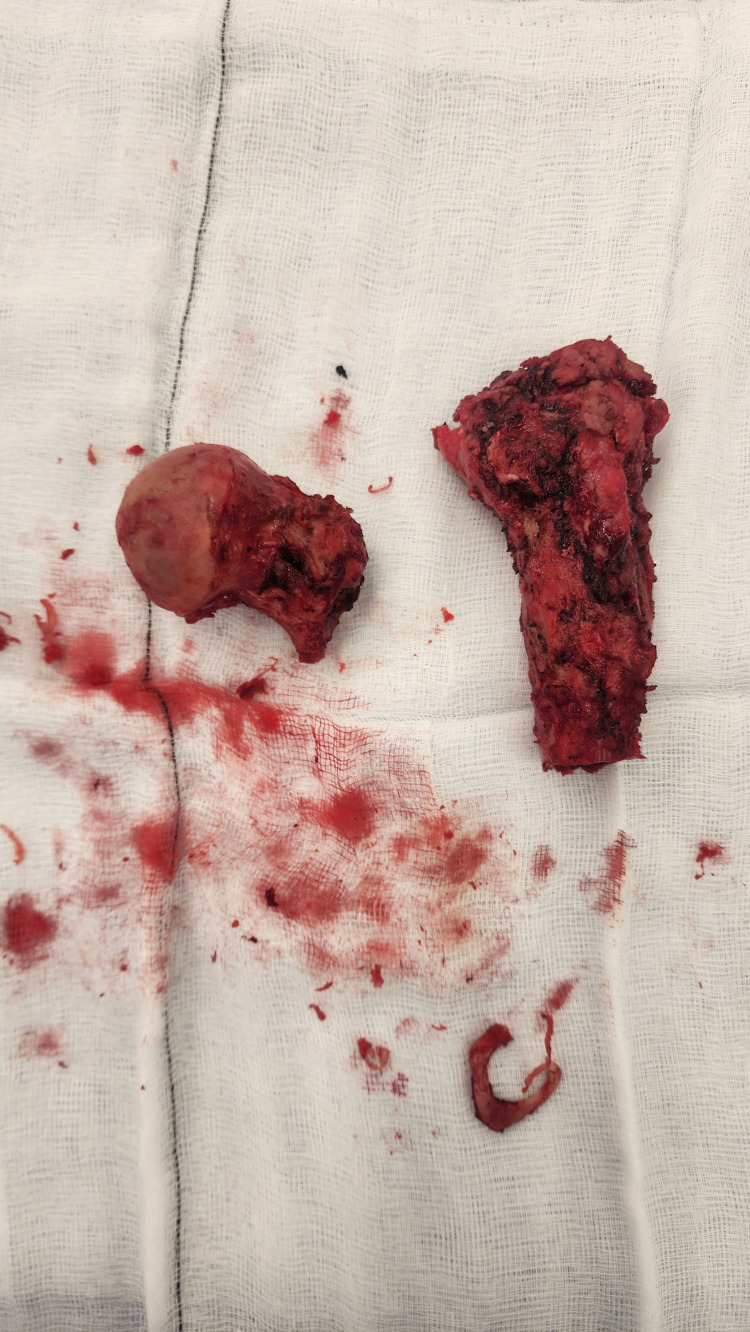
Gross appearance of resected femoral head and proximal femoral segment Intra-operative photograph displaying the resected right femoral head and proximal femoral segment side by side on a sterile field. The femoral head shows surface irregularity and subchondral changes, while the proximal segment demonstrates cortical thickening and deformity.

**Figure 5:**
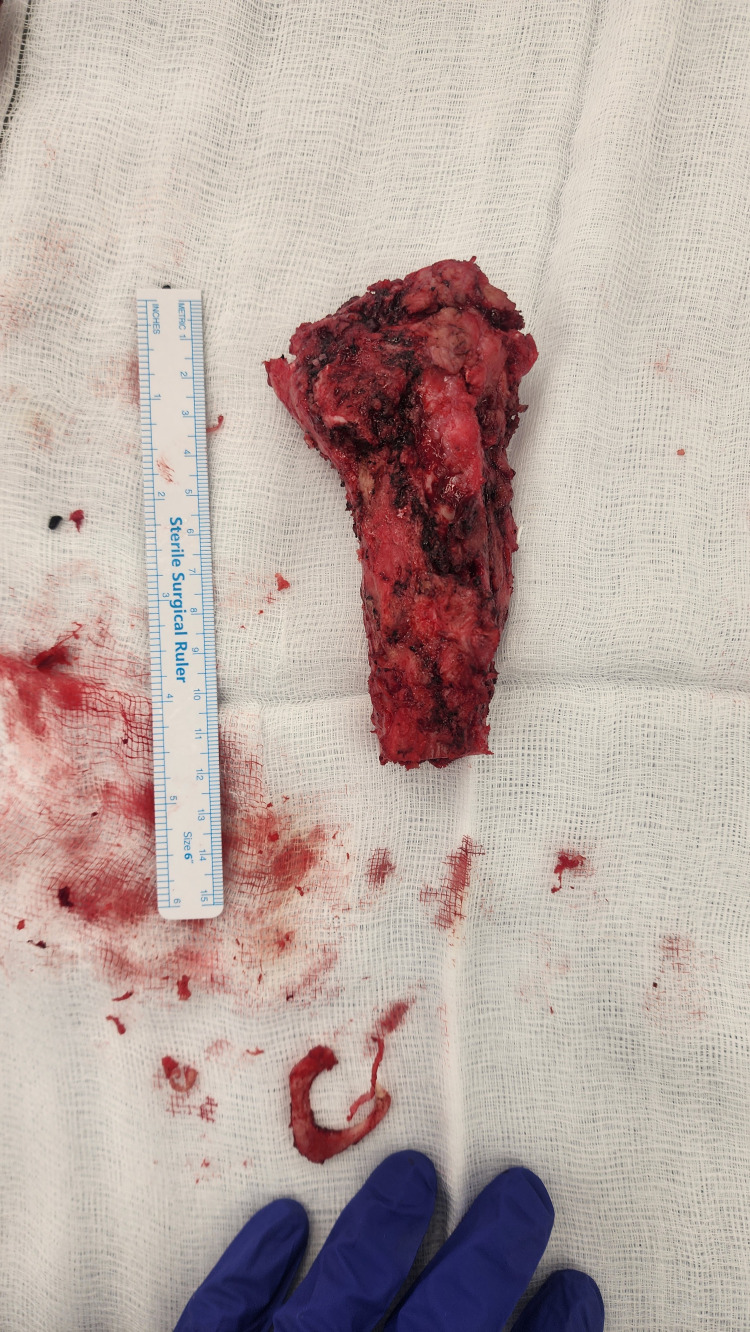
Photograph of excised right femoral segment with measuring ruler following proximal femoral replacement Intra-operative photograph showing the excised proximal femoral specimen placed on a sterile field. The specimen measures approximately 12 cm in length, and demonstrates extensive cortical irregularity and loss of normal trabecular architecture consistent with chronic pathology.

**Figure 6:**
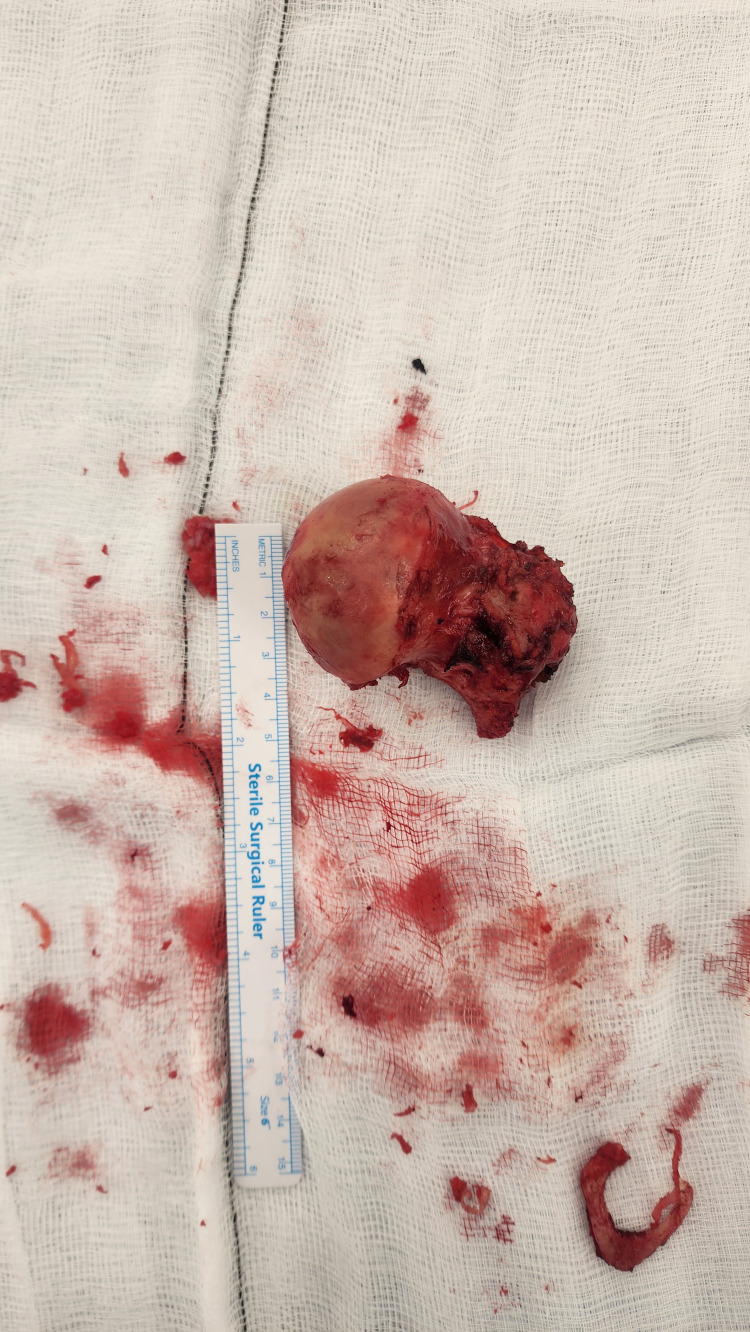
Photograph of excised right femoral head with measuring ruler following proximal femoral replacement Intra-operative photograph of the excised right femoral head placed alongside a sterile surgical ruler, demonstrating size and gross morphology. The articular surface appears irregular, reflecting degenerative and pathological changes contributing to structural compromise.

In August 2025, a cemented DePuy Synthes modular proximal femoral replacement was performed using a posterior approach (Figure 3). Along with significant anatomical distortion (Figures 4-6), the PFA was found to be trabeculated and firmly adherent along the medial femur and near the acetabular wall. Careful, sharp dissection, vascular loop isolation, intra-operative Doppler monitoring, and modified retraction techniques were employed to avoid vascular injury. An uncemented Pinnacle acetabular cup with a constrained liner was used for enhanced joint stability. The femoral stem was cemented, and antibiotic-loaded calcium sulfate (STIMULAN, Biocomposites Ltd., Staffordshire, UK) beads were placed around the implant.

**Figure 7:**
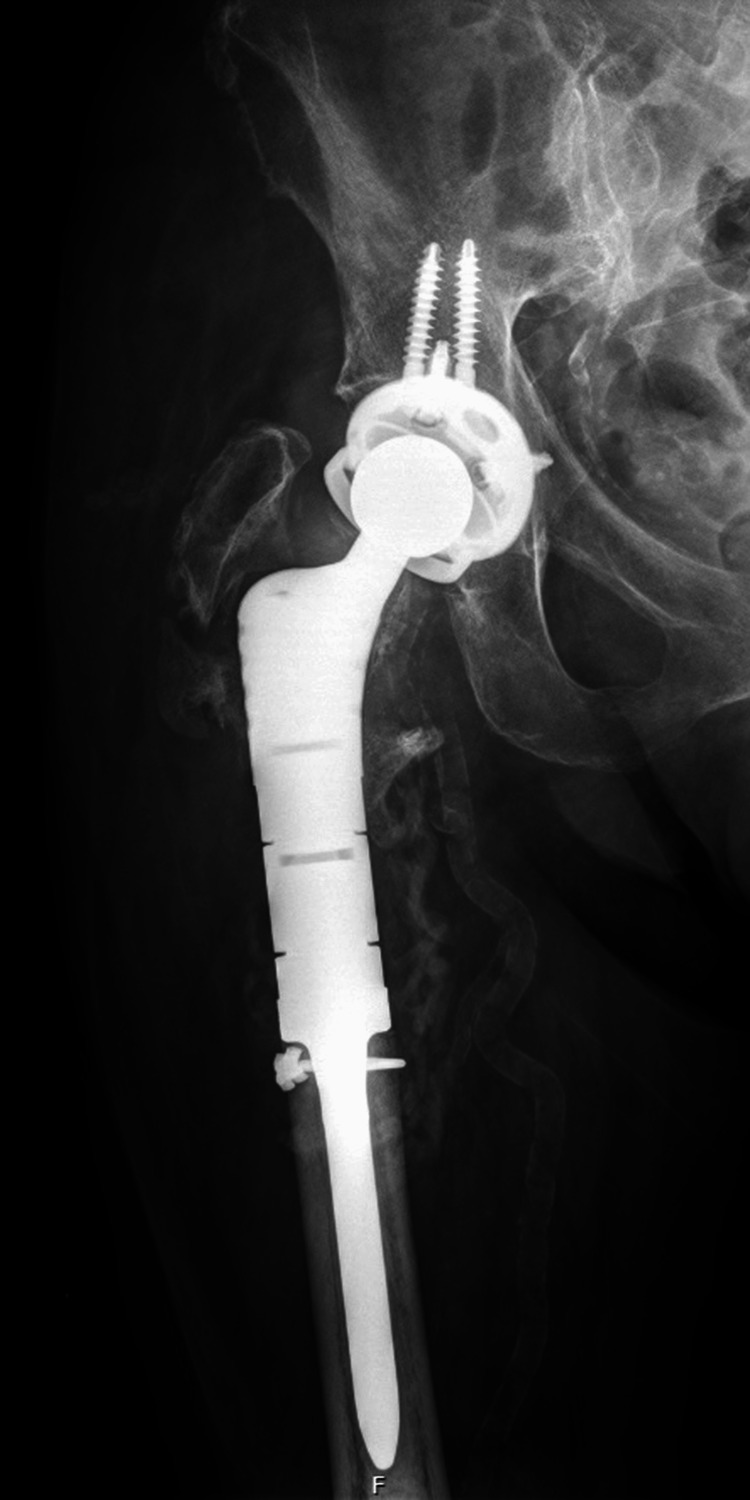
Six-week post-operative radiograph following right proximal femoral replacement Anteroposterior radiograph of the right hip and femur at six weeks post-operatively demonstrating a cemented DePuy Synthes modular proximal femoral replacement with an uncemented DePuy Synthes Pinnacle acetabular cup secured with screws. Implant alignment and positioning are satisfactory, providing a baseline for interval follow-up.

The patient was mobilized with partial weight-bearing on postoperative day 2 and discharged ambulant with a walking aid. At six-week follow-up, clinical assessment demonstrated improved right-sided lower-limb shortening, with no clinical signs of infection. Functionally, the patient mobilized indoors with a wheeled frame but required a wheelchair for outdoor mobility. Examination demonstrated an excellent range of motion. Baseline radiographs (Figure 7) confirmed satisfactory implant positioning, and further follow-up was arranged. Given the short interval since surgery, long-term outcomes relating to infection control, functional status, and implant survivorship cannot yet be assessed.

## Discussion

Management of infected non-union of the proximal femur is an orthopaedic challenge that extends beyond bone reconstruction to include infection control, restoration of function, and safeguarding of neurovascular structures. In the presence of significant bone loss and anatomical distortion from chronic infection and multiple surgeries, the reconstructive options become limited, and the surgical risk is heightened, particularly when vascular adherence complicates the field.

Several reconstructive strategies are available in such cases, including periprosthetic plating, long-stem revision arthroplasty, and PFR. Periprosthetic plating systems such as the DePuy Synthes LCP rely on good bone quality and quantity for stable fixation. However, in our patient, the proximal femoral bone was extensively debrided, osteolysed, and biologically non-viable due to previous infection. Buttaro et al. reported notable failure rates and complications after locking-plate fixation of periprosthetic femoral fractures, including hardware failure and non-union, which can significantly undermine the reliability of plating in higher-risk patients [4].

Long-stem cemented or uncemented hip revision prostheses are another consideration, but these require sufficient diaphyseal bone for adequate fixation. In chronic osteomyelitis or post-infective sequelae, this bone stock is often severely compromised. Studies by Berend et al. [5] and van Diemen et al. [6] reported substantial rates of recurrent infection and overall failure following revision for infected total hip arthroplasty.

Given these limitations, a modular cemented PFR was deemed the most appropriate solution. This approach offers multiple advantages: immediate mechanical stability, bypassing of non-viable bone, restoration of limb length and biomechanics, and facilitation of early mobilization. Furthermore, a modular megaprosthesis can provide intra-operative flexibility to restore leg length, offset, and femoral version, which is particularly valuable in cases with distorted proximal anatomy [7].

STIMULAN beads were used as an adjunct to provide high local antibiotic concentrations around the reconstruction site in the setting of previous chronic infection. This approach is supported by Jacob et al., whose systematic review of infected non-union and fracture-related infection found antibiotic-impregnated calcium sulfate to be an effective local antibiotic delivery method associated with high rates of infection eradication [8].

Despite the advantages of proximal femoral replacement, post-operative instability remains a recognized limitation, often resulting from compromised soft tissue tension and abductor dysfunction. To address this, the greater trochanter was preserved and securely sutured through the prosthesis to restore abductor mechanism integrity. Additionally, a DePuy Synthes Pinnacle acetabular component with a constrained liner was employed, leveraging evidence from Mancino et al. [9] and Hoskins et al. [10] supporting constrained liners in reducing dislocation rates and enhancing stability in complex reconstructions. Furthermore, prophylactic cerclage wiring was applied around the bone-implant interface to augment construct stability.

Beyond mechanical reconstruction, the most formidable intra-operative challenge encountered was the adherence of the superficial and profunda femoral arteries to the medial cortex of the femur and the surrounding soft tissue structures. Chronic infection-induced fibrosis, previous surgical scarring, and tissue retraction likely caused this abnormal anatomical relationship. Typically, the PFA lies in a deep, posterior location, relatively protected during standard hip exposures. However, in this case, it was found trabeculated and closely adherent to the operative field, particularly during acetabular exposure.

Injury to the PFA in this context can lead to brisk, difficult-to-control haemorrhage due to its deep course and limited accessibility. As reported by Calligaro et al. [11] and Hall et al. [12], unanticipated vascular injury during hip arthroplasty, including injury to branches of the PFA, may result in significant blood loss and compromised limb perfusion. Blind clamping in an attempt to control bleeding is dangerous and can exacerbate the injury or cause damage to adjacent structures.

Given these risks, meticulous planning and intra-operative strategy are essential. In our case, pre-operative CT angiography played a critical role in mapping vascular anatomy and identifying zones of adherence, enabling targeted surgical planning. Intra-operatively, sharp dissection, rather than blunt separation, was employed to carefully release fibrotic adhesions while minimizing traction on vascular structures. Early identification and looping of the superficial and profunda femoral arteries using vascular tapes allowed for control without excessive retraction. Handheld Doppler monitoring was used at key steps to confirm vessel patency and perfusion.

Retraction was carefully modified using malleable retractors with controlled directional pressure to avoid direct compression or shearing forces on the vessels. These adjustments were particularly important during acetabular preparation and femoral canal exposure. Although no vascular injury occurred during the procedure, preparedness was paramount. In the event of bleeding, immediate manual pressure applied over the femoral triangle remains an effective measure to temporarily occlude both the superficial and profunda branches. Hemostatic agents and field packing offer a bridge to definitive vascular control. Importantly, a vascular surgical team was alerted in advance and remained on standby throughout the procedure as a precautionary measure.

Overall, this case underscores that in the setting of complex hip reconstruction, especially with a background of chronic infection and multiple prior surgeries, PFR can serve not only as a salvage solution but as the optimal reconstructive method. The success of such procedures hinges on three pillars: appropriate implant selection based on anatomical and biological feasibility, tailored strategies to prevent instability, and, critically, a high index of suspicion and preparedness for vascular complications.

## Conclusions

Proximal femoral replacement is an effective solution in cases of complex infected non-union with massive bone loss and compromised soft tissue environments. Its use should be strongly considered when traditional fixation methods are unlikely to achieve stable, infection-free reconstruction.

However, the surgical challenge often lies not in implanting the prosthesis, but in navigating the altered anatomy, particularly when major vessels are displaced or adhered due to prior infection and fibrosis. This case underscores the importance of meticulous dissection, pre-operative vascular mapping, intraoperative Doppler use, and preparedness for vascular complications.

Proximal femoral replacement, when executed with appropriate technique and foresight, remains a vital limb-salvage tool in complex hip reconstructive surgery.
